# Diet Diversity and Adherence to a Mediterranean Diet Pattern in Pregnancy Is Protective Against the Development of Early-Childhood Atopic Dermatitis

**DOI:** 10.3390/nu17132243

**Published:** 2025-07-07

**Authors:** Kristina Nadine Heye, Leonie Helen Bogl, Mari Sasaki, Remo Frei, Anna Breunig, Neeta Bühler, Christian Raphael Kahlert, Mehmet Goekkaya, Claudia Traidl-Hoffmann, Roger Lauener, CK-CARE study group, Caroline Roduit

**Affiliations:** 1 Division of Pneumology and Allergology, Children’s Hospital of Eastern Switzerland, 9006 St. Gallen, Switzerland; 2 Department Nutrition and Dietetics, Faculty of Health Professions, Bern University of Applied Sciences, 3008 Bern, Switzerland; 3 Division of Respiratory Medicine and Allergology, Department of Pediatrics, Inselspital, University of Bern, 3010 Bern, Switzerland; 4 Christine Kühne-Center for Allergy Research and Education (CK-CARE), 7265 Davos, Switzerland; 5 Department of Biomedical Research, University of Bern, 3010 Bern, Switzerland; 6 Institute of Environmental Medicine, Helmholtz Munich—German Research Center for Environmental Health, 86156 Augsburg, Germany; 7 Institute of Environmental Medicine and Integrative Health, Faculty of Medicine, University Hospital Augsburg, 86156 Augsburg, Germany

**Keywords:** maternal diet, food frequency questionnaire, atopic dermatitis, food diversity, Mediterranean diet, pregnancy

## Abstract

Background/Objectives: The role of maternal diet in atopic dermatitis (AD) requires better understanding, as AD often manifests early in life and precedes other allergic diseases. We evaluated the association between maternal diet and AD up to 2 years of age. Methods: A total of 116 mother–child dyads from the CARE birth cohort study were included. Maternal diet during pregnancy was assessed with a validated self-administered 97-item food frequency questionnaire, and dietary scores were calculated. AD was evaluated at ages 4 months, 1 year, and 2 years. The associations between maternal dietary patterns and AD were examined by logistic regression analysis adjusting for total energy intake, gender of the child, maternal antibiotic therapy during pregnancy, and history of atopic disease among both parents. Results: Of the 116 children, 27 (23.3%) developed AD by 2 years, 11 of whom (40.7%) had persistent AD within the first 2 years. AD risk was reduced with a higher Mediterranean diet score during pregnancy (upper median > 3 points versus lower median: adjusted OR 0.24, 95% CI 0.08–0.69, *p* = 0.009) and with greater dietary diversity, as measured by the number of items consumed (upper median > 53 items versus lower median: OR 0.19, 95% CI 0.06–0.58, *p* = 0.005). No association was found with macronutrients and micronutrients. Red meat consumption showed a positive association with the persistent AD phenotype (adjusted OR 5.04, 95% CI 1.47 to 31.36, *p* = 0.034). Conclusions: Adherence to a Mediterranean diet and a diverse diet during pregnancy may decrease the risk of developing early childhood AD. This highlights the synergistic role of nutrients in dietary patterns as they modulate immune development and disease susceptibility.

## 1. Introduction

Atopic diseases are an increasing global health concern, with atopic dermatitis (AD) affecting approximately 20% of children and about 10% of adults worldwide [1]. AD often develops in infancy and is considered the first manifestation of the atopic march, particularly when the manifestation is an early-persistent phenotype, which has been associated with later development of other allergic diseases [2]. Factors involved in the etiology of AD include an epidermal barrier dysfunction with lipid abnormalities and often underlying filaggrin mutations, together with local immune dysregulation and microbial dysbiosis mediating inflammation [3]. An aberrant epidermal lipid composition and Th-2 skewed cytokine profile can be detected in infants even before the first manifestation of AD [4]. As with many chronic diseases, diet is thought to play an important role in influencing an individual’s acquisition of chronic non-communicable diseases such as allergies and AD. Among other environmental factors, maternal diet during pregnancy has the potential to influence the maturing immune system of the fetus and thereby modulate its risk of AD [5]. The proposed mechanisms linking maternal diet to offspring AD risk involve (1) the gut microbiome and (2) direct immunomodulation via vertically transferred metabolites. Prenatal maternal diet shapes the infant microbiome [6,7]. In an interventional animal study, dietary intervention during gestation with soluble fiber fed to pregnant sows changed the offspring’s intestinal microbiota composition, decreased markers of gut permeability, and increased tolerance-inducing metabolites [8]. Evidence from the Copenhagen Prospective Studies on Asthma in Childhood 2010 (COPSAC 2010) demonstrated that prenatal vertical transfer of food metabolites is associated with the risk of AD in childhood [9,10]. Some studies investigating dietary patterns have shown that a high-quality maternal diet, characterized by greater food diversity and a predominantly plant-based pattern, is protective against the development of AD in offspring [11,12,13]. In addition, adherence to a Mediterranean diet and anti-inflammatory diet in pregnancy was linked to favorable childhood respiratory allergic outcomes, however, no association with AD was reported [14,15,16,17]. On a nutrient level, an increased AD risk in the offspring has been linked to a prenatal maternal diet high in fats, including a higher *n*-6:*n*-3 fatty acid ratio [18,19,20], fast food [19], and a high-protein diet [21], while other studies found no association between maternal diet and offspring risk of AD [22,23,24,25,26].

Maternal diet offers a promising target for allergy prevention, given its modifiable nature. But findings on the association between maternal diet and childhood AD risk remain inconsistent. In this study, we aim to investigate the association between maternal nutrient intake and dietary patterns, and AD in offspring, thereby contributing to evidence-based dietary recommendations for pregnant women to promote optimal child health outcomes.

## 2. Materials and Methods

### 2.1. Study Population

The Childhood, Allergy, Nutrition and Environment (CARE) study is an ongoing birth cohort study conducted since 2016 at the Children’s Hospital of Eastern Switzerland in St. Gallen, Switzerland, as previously reported [27,28,29]. Healthy infants were prospectively recruited at the maternity ward of the Cantonal Hospital of St. Gallen, and food frequency questionnaires (FFQs) were distributed to the mothers during their stay at the maternity ward [30,31]. Information on diet was extracted from the FFQ, scores and indices calculated, and the association with AD in the offspring evaluated. Clinical follow-up visits evaluating AD including an interview and physical examination of the child by a board-certified pediatrician with special training in AD or a pediatric allergist were undertaken at ages 4 months, 1 year, and 2 years. In this study, we included 116 mother–child dyads for which both the FFQ and outcome measures were available (Supplementary Figure S1).

### 2.2. Definitions

#### Food Frequency Questionnaire (FFQ)

This FFQ has been validated in a Swiss population and assesses dietary intake in a self-administered, semi-quantitative manner, asking for the frequency and amount of 97 dietary items over the previous four weeks (Supplementary Table S1). The administration and statistical calculation of nutrients from FFQ and its validity have been well documented [30,31]. The FFQ was completed within 4 weeks after birth (peri partum period). The completeness of each FFQ was checked by a trained interviewer, and there were no missing variables in the questionnaires. The 97 different dietary items assess intake of energy, protein, fat, carbohydrates, alcohol, cholesterol, vitamin D, and retinol (>90% of its intake), and of fiber, carotene, and iron (>85% of its intake). Calculations are based on daily frequencies and reference amount measures (grams/day, except for liquids—mL/day). Intake was rated in reference to the local recommendations for pregnant women as stated by the Swiss Federal Food Office (www.blv.admin.ch, accessed on 23 July 2024). Variables from the FFQ were used to calculate predefined dietary patterns and scores, and the association with the outcome was assessed.

### 2.3. Scores and Indices

#### 2.3.1. Mediterranean Diet Score During Pregnancy

The Mediterranean diet score during pregnancy is specifically designed to assess adherence to a Mediterranean diet pattern in pregnant women. Based on traditional Mediterranean diet principles, the score includes nine food groups, each of which is assigned a score of 0 (non-adherence) or 1 (adherence) based on predefined consumption thresholds [16]. Unlike conventional Mediterranean diet scores, the consumption of dairy is rated as beneficial and alcohol as less favorable with the Mediterranean diet score during pregnancy. The total score ranges from zero to nine, with a higher score indicating greater adherence to a Mediterranean diet. As described in the original study by Chatzi et al., low adherence was defined as a total score of ≤3 points, and medium to high adherence as a total score of ≥4 points [16].

#### 2.3.2. Maternal Diet Index (MDI)

The MDI was generated as described by Venter et al. [12]. In short, the following items were rated according to frequency of intake: vegetables, yogurt, fried potatoes, rice and cooked grains (excl. pasta and bread), 100% pure fruit juice, red meats, and cold cereal. In contrast to the original score in the FFQ used, cold and cooked cereal were not differentiated. Hence, the consumption of cold cereal was possibly overestimated in our score.

#### 2.3.3. Plant-Based Diet Index (PDI)

The PDI was constructed and described by Satja et al. [32]. In short, positive scores were given to plant foods, and animal foods received negative scores. In the healthful PDI (hPDI), positive scores were assigned to fruits and vegetables, whole grains, nuts, plant oils, drinks like tea and coffee, and negative scores to less healthy plant foods including refined grains, potatoes and French fries, sweets, juices, and sweetened beverages. In the unhealthful PDI (uPDI), positive scores were assigned to less-healthy plant foods and negative scores to animal and healthy plant foods. The final individual scores potentially ranged from 0 to 60 points.

#### 2.3.4. Atopic Dermatitis

For each timepoint of clinical follow-up visit (4 months, 1 year, 2 years), AD was defined as having either (I) a diagnosis of AD during the clinical visits, (II) a doctor’s diagnosis of AD reported in the questionnaire, or (III) an itchy rash at specific locations reported by the parents in the questionnaire. Data from either clinical visits and/or questionnaires for diagnosing AD were available for 118 children at 4 months, 120 children at 1 year, and 116 children at 2 years. None of the children who were followed up until 2 years of age had more than one missing timepoint. We classified children as AD up to 1 year if they had AD at both 4 months and 1 year. We considered children with no AD for both, or no AD at only one of these timepoints, as controls. For AD up to 2 years, we identified cases as children with AD at two or more timepoints, and controls as those without AD at two or more timepoints. We classified children as controls when they had no AD at least at two timepoints and allowed for one missing timepoint or AD at one single timepoint. The latter was found in 26 children, in the majority of cases during the 4-month visit, and most diagnoses were identified by parental report of symptoms (n = 13), whereas eczema of mild severity at during the clinical visit or a diagnosis by the primary care physician was present in 12 and 3 children, respectively.

We classified children with persistent early-onset AD until 2 years if they had AD at all three timepoints and compared them to the group of children who had no AD at all three timepoints.

### 2.4. Statistical Analysis

Statistical tests were carried out in R version 4.3.2 (R Core Team 2023, R Foundation for Statistical Computing, Vienna, Austria). Non-normally distributed variables from the FFQ were log-transformed; for variables including zeroes, the log1p function was used. To determine the covariates associated with the outcomes, the two-sided Chi-square test and, for small groups, Fisher’s exact test were used for categorial variables, and for continuous variables, the simple *t*-test was applied. Logistic regression analysis was used to calculate crude odds ratios (ORs) and 95% confidence intervals (95% CIs) for associations of exposure variables extracted from the FFQ (as continuous variables), dietary patterns, and previously described scales (as quartiles and median) with AD at 4 months, until 1 year and 2 years, respectively. Logistic regression models were adjusted for total energy intake, gender, and associated baseline characteristics with the outcome (antibiotic therapy in pregnancy, atopy in family). To assess collinearity between the exposure variables that were significantly associated, Kendall’s rank correlation coefficient (τ) was used. A significance level of <0.05 was applied.

### 2.5. Pattern Analysis

Data-driven dietary patterns were assessed by PCA using 42 predefined food groups. For principal component analysis, the following packages were used: corrr, Ggcorrplot, FactoMineR, factoextraggeffects. For all variables, an orthogonal varimax rotation was applied. Retained factors had an eigenvalue greater than 2.0 and were chosen regarding their position on the inflection point in the scree plot and the interpretability of their components.

## 3. Results

### 3.1. Demographic Information

Between January 2016 and September 2021, we enrolled 122 mother–child dyads with available FFQs, of which 116 were followed up to 2 years of age (follow-up rate 95.1%; Supplementary Figure S1) and included in the final analysis. Of the total, 56 children (48.3%) were female (Table 1; results for AD at 4 months and 1 year of age are presented in Supplementary Table S2). The children were all born at term with a mean gestational age of 39.5 (standard deviation 1.8 weeks), 15 (12.9%) by cesarean section, and all were Caucasian. The vast majority of children were breastfed (95.7%) for a mean of 7.3 months. A total of 12 mothers (10.3%) were treated with systemic antibiotics during pregnancy. Moreover, 75 children (64.7%) were first-borns to the family, whereas 31 (31.9%) lived with one, 8 (6.9%) with two, and 2 (1.6%) with three older siblings. Smoking rates were observed in 10.3% of fathers and 1.7% of mothers during pregnancy. Additionally, 10.3% of mothers smoked before pregnancy. A self-reported family history of atopy was present in 83 children (71.6%). Maternal (35.3%) or paternal (34.5%) allergic rhinoconjunctivitis was most common, followed by asthma (maternal 16.4%, paternal 12.9%), AD (maternal 15.5%, paternal 10.3%), and food allergies (maternal 14.7%, paternal 9.5%). About one fifth of households (19.0%) owned a pet. The most common were cats (16.4%), followed by dogs (4.3%), and rodents owned by one family (0.9%).

### 3.2. Maternal Diet

Overall, the mothers consumed a wide range of foods and beverages, with a mean of 53 items (range 30 to 79 food items). Of the 97 items in the FFQ, 5 were not consumed: artificial sweetener/aspartame, and supplementation of vitamin C, vitamin E, fiber, and garlic extract. Local dietary recommendations during pregnancy were met by 42.2% of mothers for fruit (≥2 times per day), by 4.3% for vegetables (≥3 times per day), by 12.9% for dairy (≥3 times per day), by 34.5% for fish (≥1 times per week), and by 64.7% for meat (≤5 times per week). In total, 19 mothers (16.4%) did not meet any of the recommendations. The main source of fluid was water. The majority of mothers (87.9%) consumed sweetened drinks (excluding milk). Bottled fruit juice was more often consumed than fresh fruit juice (bottled juice in 50.5% versus fresh juice in 33.1% of mothers).

#### 3.2.1. Macronutrients

**Figure 1:**
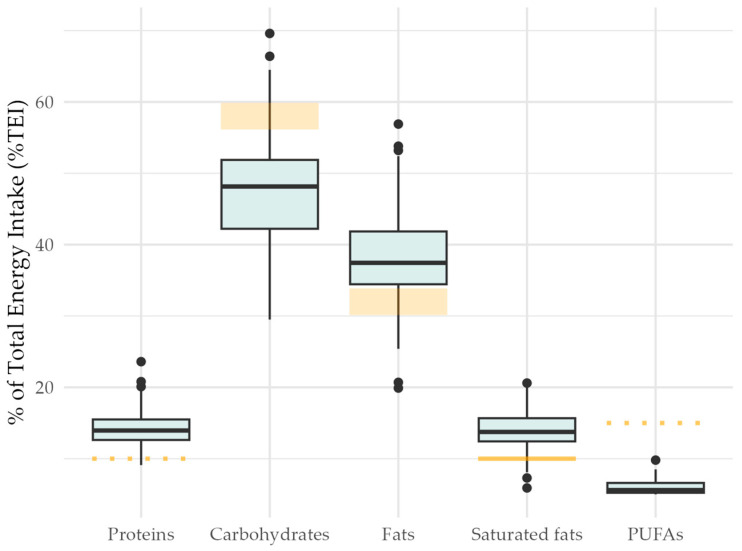
Macronutrient contributions to total energy intake. Percentages of macronutrient intake of total energy intake (TEI) are presented as boxplots with median and interquartile range (IQR). Orange areas represent local recommendations: proteins > 10%TEI (dotted line), carbohydrates 55–60%TEI (area), fats 30–35%TEI (area) with saturated fats < 10%TEI (solid line), and polyunsaturated fats (PUFAs) > 15%TEI (dotted line).

Of the 116 mother–child dyads, three mothers (2.6%) followed a vegetarian diet. Overall, protein sources were mainly animal proteins as indicated by a median ratio of vegetal to animal protein of 0.51 (interquartile range 0.38 to 0.70). Primarily, plant-based protein intake (ratio of plant to animal protein > 1.0) was found in 11 mothers (9.5%). The consumption of red meat and white meat was variable, with an average consumption of 41 g per day (ranging 4 to 178 g) and 28 g per day (ranging 1 to 95 g), respectively. A quarter (26.1%) of consumed meat was processed meat like sausages. Carbohydrate intake of nearly two thirds of mothers (60.3%) was according to the local recommendations of 45–60% of total energy intake (TEI), and monosaccharides and polysaccharides were found to be equally consumed with 22% and 23% of TEI, respectively (Figure 1). The average total fat consumption was found to be on the upper limit. The consumption of saturated fat was greater than the recommended maximal 10% TEI in 108 mothers (93.1%) and exceeded the recommendation up to twofold. Saturated fats were consumed in smaller amounts than polyunsaturated fats, as reflected in an overall ratio of saturated to unsaturated fats of 0.69 (standard deviation 0.13).

#### 3.2.2. Micronutrients, Minerals, and Fiber

Dietary intakes of vitamin D and iron were 1.79 micrograms per day (standard deviation 1.24) and 9.65 micrograms per day (standard deviation 3.44), respectively. This is below the recommended intake of pregnant women and highlights the importance of vitamin supplementation to achieve the recommended intake of 15 micrograms of vitamin D and 30 milligrams of iron per day. The average fiber intake was below the recommendations. Eleven mothers (9.5%) achieved the recommended intake of 30 g/day.

### 3.3. AD in the Child

AD was present in 27 children (23.3%) until 2 years, and 11 children (9.5%) had a persistent AD pattern defined as all three timepoints presenting with AD. AD was of mild or moderate severity. None presented with severe eczema. Median SCORAD scores at 2 years of age were 18.0 points (range 3.7 to 24.0, 4 moderate).

### 3.4. Association of Maternal Diet with AD in the Child

**Figure 2:**
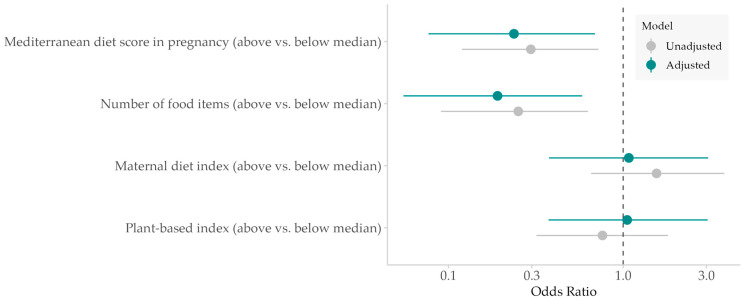
Association of maternal diet indices with atopic dermatitis in the child. Regression models for AD with median split exposure variables adjusted for total energy intake, gender, atopic disease in the parents, and antibiotic therapy in pregnancy.

The association between AD and dietary indices by quartiles revealed the most pronounced effects in the upper quartiles (Q3 and Q4) versus the lowest (Supplementary Table S3). Consequently, dietary exposures were dichotomized at the median for further analysis. Figure 2 presents the results of logistic regression models estimating the effect of maternal dietary patterns on the risk of AD development in offspring until 2 years of age. A Mediterranean diet score in pregnancy greater than 3 points (corresponding to the median) was associated with a reduced risk of AD in offspring (crude OR 0.30, 95% CI 0.12–0.72, *p* = 0.007; adjusted OR 0.24, 95% CI 0.08–0.69, *p* = 0.009). Greater food diversity reflected by >53 food items consumed (upper median) compared to a lower number of food items consumed was protective for AD in the child (crude OR 0.25, 95% CI 0.09–0.63, *p* = 0.005; adjusted OR 0.19, 95% CI 0.06–0.58; *p* = 0.005). A very weak positive association was found between these two exposure variables (Kendall’s τ = 0.14, *p* = 0.042), deemed not practically significant. Given the small effect size, this relationship may not be practically meaningful. Additionally, we performed a sensitivity analysis by excluding the 26 children with AD at only one single timepoint from the control group and found similar results (Supplementary Table S5). The MDI (median > 71.3, IQR 70.46–72.06 points) and the PDI (median > 15, IQR 12–21 points) were not significantly associated with the outcome for any of the three timepoints. However, there was a trend of harmful effects of higher unhealthy PDI scores (median > −22.1 points) for AD until 2 years of age (crude OR 2.33, 95% CI 0.97–5.96, *p* = 0.065; adjusted OR 2.96, 95% CI 0.92 to 10.42; *p* = 0.075).

There were no associations between offspring AD and individual macronutrient or micronutrient intakes (Table 2 and Supplementary Table S4). The only association found with the persistent early-onset AD phenotype up to 2 years of age (11 children with AD at all three timepoints) versus 50 healthy controls (no AD throughout all three timepoints) was with and increased maternal red meat consumption (crude OR 3.2, CI 1.30 to 11.3, *p* = 0.034, adjusted OR of 5.04, 95% CI 1.47 to 31.36, *p* = 0.034), and trendline with a plant-protein based diet (plant-to-animal-protein ratio; crude OR 0.23, 95% CI 0.04 to 1.01, *p* = 0.070, adjusted model: OR 0.15, CI 0.01 to 0.98, *p* = 0.077).

We used PCA to identify clusters of foods eaten together, simplify the complexity of the dietary measures, and reflect actual eating behavior. The total explained variances of the dietary patterns were small, with 11.6% for pattern 1 (healthy, high-fat dairy) and 7.6% for pattern 2 (red meat, low in plant proteins, sweets). A healthy, high-fat dairy diet was trendline protective for AD up to 1 year but did not reach significance (adjusted OR 0.77, 95% CI 0.55–1.05, *p* = 0.087; Supplementary Table S6). The other dietary pattern did not show any association with AD at any of the three timepoints. The correlations of food groups for the identified patterns are presented in Supplementary Tables S7 and S8.

## 4. Discussion

In this birth-cohort, adherence to a Mediterranean diet and greater diet diversity during pregnancy were associated with a reduced risk of AD in the child up to 2 years of age. In contrast, single macronutrients and micronutrients were not associated with the outcome.

The effect of the Mediterranean diet in pregnancy and offspring atopic diseases has been studied in late-preschool-age and school-age children, and a negative association has been observed with atopic wheeze and better small airway function, but not with AD [16,19,33]. Focusing on AD in younger children, we demonstrate that adherence to a Mediterranean diet pattern was protective. Characterized by high intake of olive oil, plant-proteins, fruit, and fish, and limited amounts of red meat, numerous health benefits have been attributed to this diet pattern, which is particularly rich in fibers and monosaturated and polyunsaturated fatty acids [34,35]. It has been suggested that a Mediterranean diet reduces allergic disease risk in children, possibly via modulation of immune responses and promotion of microbiome health through increased microbial diversity and anti-inflammatory effects of bioactive compounds present in this diet [34]. Healthy fats, including MUFAs from olive oil, possess anti-inflammatory properties that may modulate immune responses involved in atopic conditions [36]. Soluble fibers and long-chain polysaccharides like beta-glucans and inulins undergo fermentation by gut microbiota, leading to the production of short-chain fatty acids (SCFAs) such as butyrate, propionate, and acetate [37]. SCFAs can regulate inflammation and a vital mediators for maintaining the epithelial barrier health in the skin, the gut, and the respiratory tract [38,39]. In the skin, SCFAs induce anti-inflammatory cytokines, including IL-10, and regulatory T-cells (Tregs), and modulate mitochondrial metabolism of epidermal keratinocytes, thereby enhancing the integrity of the epithelial barrier [39,40,41]. We showed previously that disrupted gut microbiome development dependent on SCFA-producing bacteria increases the risk of AD [28]. However, we did not find an association between butyrate-promoting foods such as fruits, vegetables, high-fat dairy products, and fiber and AD in this study, but the effect sizes of individual foods or nutrients might be too small to detect.

High dietary quality with a variety of foods in pregnancy together with infancy diet may potentiate the protective effect for allergy development [42]. We observed a negative association between a higher number of different food items consumed during pregnancy and AD in children. Similar results have been shown in a Swedish birth cohort study measuring the diet diversity score in the third trimester of pregnancy [11]. A higher diet diversity was protective for AD in the offspring at 18 months, with a 43% lower risk for moderate AD in the highest quartile compared with the lowest quartile. Likewise, a negative association between healthy diet diversity in pregnancy and allergy in children was evident in the Healthy Start study [12]. Diet diversity supports a healthy microbiome in both adults and young children, and a protective effect for allergic disease in the latter has been shown [43,44]. A detrimental effect on the child’s microbiome has been demonstrated for a maternal diet high in fats, while fruit consumption seemed supportive for microbiome health [45,46]. We found a positive association between maternal red meat consumption and persistent AD phenotype, and a trend for a protective effect of a mainly plant protein-based diet. This is in line with the finding of a negative association of a plant-based diet with AD at 1 year of age in one study from the NutriGen Alliance [13]. In a prospective cohort study, Li et al. found a trend for increased risk of AD at 6 and 12 months of age when mothers consumed a high-protein diet during pregnancy [21]. However, the protein source was not differentiated in this study. In contrast, Chatzi et al. found that high and processed meat intake were associated with an increased risk of wheezing at 1 year, but not with AD [22]. These findings underscore the potential importance of maternal protein sources during pregnancy, suggesting that higher intake of plant-based proteins, as opposed to red or processed meats, may be associated with a reduced risk of early-life atopic outcomes including AD. Furthermore, a healthy maternal diet is a direct and indirect promoter of the gut ecosystem health, priming immune cells in utero and supporting the diversity and maturation of intestinal bacteria [7]. While a delayed gut microbiome development increases the risk of allergy, this modulation of the immune system by food components and their fermentation products can potentially alter the evolution of the atopic trajectory [47].

We found no associations between individual macronutrients or micronutrients and AD. As different foods are often consumed together rather than in isolation, the importance of a synergistic effect between the different foods and their components has been suggested and may explain the lack of associations with single foods or nutrients in our study [48]. Unlike individual nutrients, which may have limited or inconsistent effects when evaluated independently, comprehensive dietary patterns involve complex interactions that can either enhance or attenuate their influence on immune development and disease susceptibility [49,50]. Several mechanisms evidence the synergistic effects of dietary patterns. Some examples include (1) polyphenols from fruits and vegetables amplifying the anti-inflammatory effects of Omega-3 fatty acids from fish (antioxidant interaction), combined effects of fermented products, fiber-rich foods, short-chain fatty acids, and polyphenol-containing foods support a beneficial gut microbiota, which is essential for immune tolerance and reduced risk of atopy (gut microbiome modulation), (2) facilitated absorption of fat-soluble vitamins that are enhanced by the presence of polyphenols in the diet, which is essential for immune tolerance and reduced risk of atopy (metabolism), (3) facilitated absorption of fat-soluble vitamins, which is enhanced by the presence of monosaturated and polyunsaturated fats (metabolism) and epigenetic modifications [51,52,53]. All of the above principles are reflected in the Mediterranean diet [54]. In the FFQ used, the distinction between Omega-3 and Omega-6 fatty acids was not made, with the latter having a potentially opposite effect. Therefore, the protective aspect of Omega-3 fatty acids in the Mediterranean diet can only be hypothesized but not confirmed in this study.

Moreover, our finding of an inherited risk for acquiring AD is consistent with previous research confirming a strong genetic predisposition to atopic diseases and well-defined target genes, such as filaggrin mutations in AD [55]. In addition, known risk factors for atopic disease, including mode of delivery, lifestyle (urban, older siblings), and antibiotic therapy, may impede microbiota expansion and increase the risk for allergic disease in the child [47]. Interestingly, we found that antibiotic therapy during pregnancy had a harmful effect on the development of AD in the child, while therapy throughout the first year of life had no influence.

The strengths of this study are its prospective design and repeated clinical assessments for AD. The FFQ was applied during the peripartum period, limiting recall bias. Nevertheless, our study has several limitations. We found a high prevalence of atopic diseases in family history and AD in the offspring. We suspect that this is due to selection bias. To take the transient and mild character of AD into account, we classified children with missing values at one timepoint or AD at one single timepoint as controls. Aware that this definition could potentially lead to the misclassification of the controls, we performed a sensitivity analysis and found no differences in the key findings. A limitation of the study is the assessment of exposure by FFQ at a single timepoint, reflecting the diet during the perinatal period. This is a time during pregnancy when dietary choices may be more selective due to pregnancy symptoms [56]. In addition, information on maternal body mass index, weight gain, and exercise during pregnancy was not available. Exploring the association of maternal diet measures with the persistent AD phenotype subgroup, several exposure variables may not have reached significance, and some logistic regression intervals were relatively wide, including the meat-and-protein ratio, reflecting imprecision in our estimates, likely driven by the sample size. Additionally, a limitation is that only limited postnatal child dietary data were included in the study, but no measures such as diet diversity or detailed nutrient intake, as these data were not available.

## 5. Conclusions

In conclusion, maternal adherence to a Mediterranean diet pattern, a diverse diet, and restrained red meat consumption during pregnancy may be protective against the development of early-onset AD in offspring. Prenatal antibiotic therapy was another potentially modifiable harmful factor. However, the mechanisms through which maternal diet and environmental factors influence maternal health and offspring atopic diseases are complex, and more research is needed to understand these interactions and their scale.

## Figures and Tables

**Table 1:** Characteristics.

	Overall n = 116	Controls	AD 2 Years	
	n (%), Mean (sd)	89 (76.7%)	27 (23.3%)	*p-Value* (Healthy vs. AD)
Female, n (%)	56 (48.3)	45 (50.6)	11 (40.7)	*0.500*
Antibiotic therapy during pregnancy, n (%)	12 (10.3)	4 (4.5)	8 (29.6)	***0.001***
Antibiotic therapy in child up to 4 months, n (%)	7 (6.0)	4 (4.5)	3 (11.1)	*0.351*
Antibiotic therapy in child up to 1 year, n (%)	17 (14.7)	11 (12.4)	6 (22.2)	*0.338*
Cesarean section, n (%)	15 (12.9)	12 (13.5)	3 (11.1)	*1.000*
Mother avoided food (during pregnancy), n (%)	6 (5.2)	5 (5.6)	1 (3.7)	*1.000*
Mother avoided food at 4 months, n (%)	11 (9.8)	7 (8.0)	4 (16.0)	*0.426*
Child breastfed at any timepoint, n (%)	110 (95.7)	86 (96.6)	24 (92.3)	*0.686*
Weaning, (months), mean (sd)	7 (2.9)	7 (2.8)	8 (3.1)	*0.178*
Introduction of solids (wks), mean (sd)	19.8 (2.9)	19.7 (3.0)	20.0 (2.6)	*0.668*
Any siblings, n (%)	41 (35.3)	35 (39.3)	6 (22.2)	*0.162*
Pet (cat, dog, bird, rodent), n (%)	22 (19.0)	16(18.0)	6 (22.2)	*0.832*
Mother and father atopic disease, n (%)	28 (24.1)	17 (19.1)	11 (40.7)	***0.041***

*p*-values < 0.05 in bold, statistical test used for group comparison: chi-square (small groups: Fisher’s exact test) and *t*-test.

**Table 2:** Association of macronutrients and micronutrients with atopic dermatitis in the child.

		Atopic Dermatitis Until 2 Years	
	n = 116	n = 27 (23.3%)	
	Mean (SD), Median (Q1, Q3), n (%)	OR (95% CI)	*Adjusted* *p-Value*
**Maternal diet**			
**Macronutrients in %TEI**			
Total protein	13.95 [12.67, 15.50]	0.60 (0.04–9.31)	*0.714*
Vegetal-to-animal-protein ratio	0.51 [0.38, 0.70]	0.96 (0.16–4.53)	*0.958*
Total carbohydrates	48.15 [42.15, 52.40]	0.69 (0.04–13.66)	*0.805*
Total fats	37.40 [34.20, 42.00]	1.43 (0.09–25.88)	*0.803*
Saturated fat (SFA)	14.03 (2.83)	1.04 (0.88–1.23)	*0.655*
Monounsaturated fat (MUFA)	15.81 (3.72)	1.03 (0.89–1.18)	*0.696*
Polyunsaturated fat (PUFA)	4.90 [4.20, 5.60]	0.93 (0.61–1.38)	*0.738*
**Fiber and Micronutrients**			
Total fiber, gr	15.13 [10.64, 20.35]	1.36 (0.32–6.12)	*0.678*
Iron, mg	9.65 (3.44)	3.25 (0.16–83.36)	*0.458*
Retinol	338.42 (225.83)	1.00 (1.00–1.01)	*0.220*
Vitamin D	1.79 (1.24)	1.14 (0.54–2.42)	*0.733*
Daily multivitamins	74 (63.8)	2.47 (0.84–8.56)	*0.121*
Sweetened drinks, mL	70.54 [25.00, 210.27]	0.95 (0.73–1.25)	*0.698*
Water consumption, mL	771.15 (325.90)	1.01 (0.66–1.81)	*0.967*
**Food groups**			
Fruit portions/day	1.43 [0.91, 2.68]	1.12 (0.34–3.48)	*0.849*
Vegetable portions/day	1.48 [1.01, 2.13]	0.79 (0.17–3.62)	*0.766*
Any nut and soy consumption	23 (19.8)	1.96 (0.59–6.20)	*0.255*
Quantity of red meat, g/day	41.07 [16.63, 58.08]	1.10 (0.73–1.77)	*0.668*
Quantity of white meat, g/day	27.68 [13.79, 40.45]	0.67 (0.41–1.07)	*0.094*
Fish, g/day	13.39 [5.36, 25.11]	0.80 (0.55–1.18)	*0.206*
Dairy products, g/day *	2.43 [1.67, 3.34]	0.55 (0.10–3.17)	*0.502*

Regression models are adjusted for total energy intake, gender, atopic disease in the parents, and antibiotic therapy in pregnancy. * Dairy products including butter and cream.

## Data Availability

The original data presented in the study are openly available in the Bern Open Repository and Information System (BORIS) at https://doi.org/10.48620/88411.

## Supplementary Materials

The following supporting information can be downloaded at: https://www.mdpi.com/article/10.3390/nu17132243/s1. Supplementary Figure S1. Flowchart of the study cohort; Supplementary Table S1. Food items included in the food frequency questionnaire (FFQ); Supplementary Table S2. Baseline characteristics and atopic dermatitis in the child at 4 months and until 1 year of age; Supplementary Table S3. Association of diet indices with atopic dermatitis in the child; Supplementary Table S4. Association of macronutrients and micronutrients with AD in the child at 4 months and until 1 year of age; Supplementary Table S5. Sensitivity analysis of controls by transient AD status; Supplementary Table S6. PCA for individual scores and association with atopic dermatitis in the child; Supplementary Table S7. Representative variables of a given principal component (pattern); Supplementary Table S8. Correlation of food groups with PCA-derived patterns.
