# Supplementary material for: Diet Diversity and Adherence to a Mediterranean Diet Pattern in Pregnancy Is Protective Against the Development of Early-Childhood Atopic Dermatitis
Source: Nutrients. 2025 Jul 7;17(13):2243. doi: 10.3390/nu17132243 (PMC12252201; doi:10.3390/nu17132243)
Supplement: Supplementary file 1 [file nutrients-17-02243-s001.zip › nutrients-3729696-supplementary.pdf]

Supplementary Figure S1. Flow-chart of the study cohort

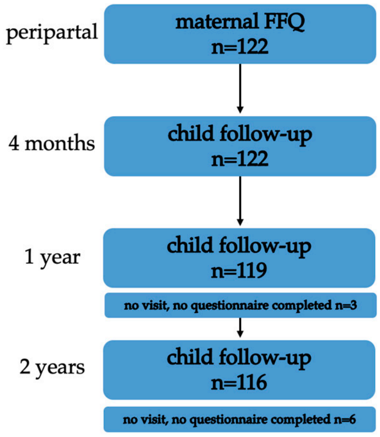

Supplementary Table S1. Food items included in the food frequency questionnaire (FFQ)

1	Plain yogurt
2	Low-fat yogurt
3	Fruit/aroma yogurt
4	Cottage cheese (0% fat)
5	Cottage cheese, ricotta
6	Feta, mozzarella
7	Gruyère cheese, Camembert cheese
8	Cheese fondue
9	White or soft bread, tresse
10	Whole wheat bread, rye bread
11	Cold cereal, porridge
12	Corn-flakes, puffed rice
13	Rusks, Swedish bread
14	Beef, horse, veal
15	Chicken, skinned
16	Chicken, with skin
17	Hamburger, rib steak, roasted meat (Beef, horse or veal)
18	Cured ham, mutton or pork chops
19	Sausage, salami, ham
20	Pâté, terrine
21	Cervelas, wieners
22	Frankfurter, small sausages
23	Liver (veal or pork)
24	Liver (poultry)
25	Salmon (fresh or smoked)
26	Fried or breaded fish
27	Tuna in oil
28	White fish (cod, trout, hake)
29	Seafood (shrimps, mussels)
30	Green beans, spinach
31	Cauliflower, broccoli
32	Tomatoes
33	Carrots
34	Green salad
35	Vinaigrette sauce
36	Green peas, corn, maize
37	Avocado
38	Vegetable broth
39	Vegetable soup (peas, beans, minestrone)
40	Potatoes, boiled
41	French fries
42	Pasta
43	Ravioli, tortellini, cannelloni
44	Rice
45	Tomato sauce
46	Couscous, semolina
47	Pizza
48	Quiche
49	Eggs (any processing)
50	Tofu
51	Margarine, low fat
52	Butter

53	Cream (35% fat)
54	Mayonnaise sauce
55	Banana, apple, pear, plum, grapes
56	Citrus fruits (orange, tangerine)
57	Peach, apricot, melon
58	Berries (strawberries, blueberries)
59	Kiwi
60	Fruit, preserved
61	Croissant, chocolate bread
62	Fruit tart
63	Cream tart, cream cake
64	Cake, dried pastries
65	Biscuits, cookies
66	Chocolate
67	Honey, jam
68	Ice cream, sorbet
69	Artificial sweeteners (aspartame)
70	Sugar
71	Butter (for cooking)
72	Margarine (for cooking)
73	Olive oil (for cooking)
74	Peanut oil (for cooking)
75	Sunflower seed oil (for cooking)
76	Vitamin C supplements
77	Vitamin E supplements
78	Multivitamin supplements
79	Dietary fiber supplements
80	Garlic extract dietary supplements
81	Coffee (excl. Decaffeinated)
82	Milk in coffee (0% fat)
83	Milk in coffee (non 0% fat)
84	Coffee creamer
85	Milk as drink (0% fat)
86	Milk as drink (non 0% fat)
87	Mineral water (Aproz, Valser, San Pellegrino)
88	Mineral water (Perrier, Vittel, Volvic)
89	Tap water, mineral water (Henniez, Evian, Vichy)
90	Lemonade, soda, syrup
91	Fresh fruit juice
92	Bottled fruit juice
93	Tea, herbal tea
94	Beer
95	Wine, champagne
96	Aperitifs (Martini)
97	Spirits (whisky, liquor)

Supplementary Table S2. Baseline characteristics and atopic dermatitis in the child at 4 months and until 1 year of age

	**4 months**			**1 year**		
	Control	AD	*Adj. p-value*	Control	AD	*Adj. p-value*
Children, n	87	29		100	16	
Female	45 (51.7)	11 (37.9)	*0.283*	49 (49.0)	7 (43.8)	*0.904*
Antibiotic therapy during pregnancy	5 (5.7)	7 (24.1)	***0.014***	7 (7.0)	5 (31.2)	***0.012***
Antibiotic therapy in child up to 4 months	4 (4.6)	3 (10.3)	*0.499*	5 (5.0)	2 (12.5)	*0.546*
Antibiotic therapy in child up to 1 year				13 (13.0)	4 (25.0)	*0.379*
Caesarean section	9 (10.3)	6 (20.7)	*0.263*	13 (13.0)	2 (12.5)	*1.000*
Any siblings	33 (37.9)	8 (27.6)	*0.432*	38 (38.0)	3 (18.8)	*0.225*
Pet (cat, dog or rodent)	16 (18.4)	6 (20.7)	*1.000*	17 (17.0)	5 (31.2)	*0.314*
Mother avoided food (during pregnancy)	4 (4.6)	2 (6.9)	*1.000*	5 (5.0)	1 (6.2)	*1.000*
Mother avoided food (at 4 months)	8 (9.4)	3 (11.1)	*1.000*	8 (8.2)	3 (21.4)	*0.28*
Child avoided food (at 1 year)	15 (17.6)	7 (25.0)	*0.564*	17 (17.3)	5 (33.3)	*0.269*
Child breastfed at any timepoint	84 (96.6)	26 (92.9)	*0.763*	97 (97.0)	13 (86.7)	*0.25*
Weaning, months	7 (3)	9 (2)	*0.057*	7 (3)	9 (1)	*0.133*
Introduction of solids (weeks)	20.05 (2.87)	18.96 (2.96)	*0.088*	19.83 (3.03)	19.47 (2.07)	*0.655*
Bathing min. every 1 to 3 days (4 months)	*17 (19.5)*	*12 (41.4)*	***0.035***	*23 (23.0)*	*6 (37.5)*	*0.351*
Using soap when bathing (4 months)	*41 (47.1)*	*19 (65.5)*	*0.133*	*52 (52.0)*	*8 (50.0)*	*1.000*
Bathing min. every 1 to 3 days (1 year)*				*36 (36.4)*	*11 (73.3)*	***0.015***
Using soap when bathing (1 year)*				*75 (75.0)*	*6 (40.0)*	***0.014***
Mother and father atopic disease	17 (19.5)	11 (37.9)	*0.079*	*23 (23.0)*	5 (31.2)	*0.688*

p-values < 0.05 in bold, statistical test used: chi-square (small groups: Fisher's exact test), t-test
AD, atopic dermatitis
* In line with recommendations for AD skin care.

Supplementary Table S3. Association of diet indices with atopic dermatitis in the child

		**Atopic dermatitis 2 years** 23.3% (n=27/116)	
		**OR (95% CI)**	*adjusted p-value*
**Maternal diet scores and indexes**			
Mediterranean Diet Score during Pregnancy	Reference Q1		
	Q2 vs Q1	0.36 (0.11–1.14)	*0.088*
	Q3 vs Q1	0.09 (0.01–0.45)	***0.008***
	Q4 vs Q1	0.21 (0.02–1.21)	*0.111*
Number of food items consumed	Reference Q1		
	Q2 vs Q1	1.05 (0.3–3.67)	*0.938*
	Q3 vs Q1	0.22 (0.04–1.02)	*0.067*
	Q4 vs Q1	0.22 (0.04–0.96)	*0.056*
Maternal Diet Index MDI Score	Reference Q1		
	Q2 vs Q1	0.54 (0.11–2.52)	*0.441*
	Q3 vs Q1	0.8 (0.17–3.48)	*0.766*
	Q4 vs Q1	0.91 (0.19–4.3)	*0.907*
Plant-based index (PDI)	Reference Q1		
	Q2 vs Q1	0.8 (0.21–3.02)	*0.739*
	Q3 vs Q1	0.84 (0.21–3.19)	*0.797*
	Q4 vs Q1	0.7 (0.14–3.25)	*0.653*

Regression models are adjusted for total energy intake, gender, atopic disease in the parents, and antibiotic in pregnancy.

Supplementary Table S4. Association of macronutrients and micronutrients with AD in the child at 4 months and until 1 year of age

	n=116	Atopic dermatitis at 4 months n=29 (25.0 %)		Atopic dermatitis until 1 year n=16 (13.8%)	
	mean (SD), median (Q1, Q3), n (%)	**OR (95% CI)**	***adj. p-value***	**OR (95% CI)**	***adj. p-value***
**Maternal diet**					
**Macronutrients %TEI**					
Total protein	13.95 [12.67, 15.50]	0.82 (0.06–12.24)	*0.886*	9.62 (0.34–361.10)	*0.197*
Ratio vegetal to animal protein	0.51 [0.38, 0.70]	0.67 (0.12–2.96)	*0.623*	0.53 (0.05–3.62)	*0.566*
Total carbohydrates	48.15 [42.15, 52.40]	0.16 (0.01–2.65)	*0.204*	0.78 (0.03–26.51)	*0.889*
Total fats	37.40 [34.20, 42.00]	3.12 (0.23–50.93)	*0.407*	0.37 (0.02–9.22)	*0.534*
Saturated fat (SFA)	14.03 (2.83)	1.11 (0.94–1.32)	*0.216*	1.04 (0.85–1.27)	*0.713*
Monounsaturated fat (MUFA)	15.81 (3.72)	1.09 (0.96–1.24)	*0.205*	0.94 (0.79–1.10)	*0.479*
Polyunsaturated fat (PUFA)	4.90 [4.20, 5.60]	0.93 (0.62–1.36)	*0.719*	0.67 (0.35–1.14)	*0.173*
**Fiber and Micronutrients**					
Total fiber, gr	15.13 [10.64, 20.35]	1.53 (0.39–6.28)	*0.546*	0.72 (0.13–3.88)	*0.694*
Iron, mg	9.65 (3.44)	1.92 (0.12–36.7)	*0.653*	5.42 (0.15–288.41)	*0.378*
Retinol	338.42 (225.83)	1.00 (1.00–1.01)	*0.392*	1.00 (1.00–1.01)	*0.153*
Vitamin D	1.79 (1.24)	0.61 (0.28–1.29)	*0.205*	0.74 (0.29–1.80)	*0.511*
Daily multivitamins	74 (63.8)	0.96 (0.37–2.53)	*0.925*	2.3 (0.65–10.31)	*0.227*
Sweetened drinks, mL	70.54 [25.00, 210.27]	1.06 (0.82–1.41)	*0.652*	1.09 (0.78–1.61)	*0.641*
Water consumption, mL	771.15 (325.90)	1.02 (0.67–1.84)	*0.937*	0.89 (0.57–1.83)	*0.657*
**Food groups**					
Fruit portions / day	1.43 [0.91, 2.68]	1.33 (0.45–3.79)	*0.597*	0.43 (0.10–1.63)	*0.236*
Vegetable portions / day	1.48 [1.01, 2.13]	0.73 (0.17–2.97)	*0.657*	0.37 (0.06–2.04)	*0.264*
Any nut and soy consumption	23 (19.8)	0.96 (0.29–2.90)	*0.946*	1.51 (0.35–5.63)	*0.551*
Quantity of red meat, g/day	41.07 [16.63, 58.08]	1.39 (0.90–2.33)	*0.169*	1.47 (0.84–3.12)	*0.239*
Quantity of white meat, g/day	27.68 [13.79, 40.45]	0.88 (0.56–1.42)	*0.590*	0.95 (0.54–1.82)	*0.863*
Fish, g/day	13.39 [5.36, 25.11]	0.89 (0.62–1.29)	*0.527*	0.94 (0.62–1.47)	*0.769*
Dairy products, g/d*	2.43 [1.67, 3.34]	1.2 (0.23–6.50)	*0.829*	0.52 (0.06–4.19)	*0.539*

Regression models are adjusted for total energy intake, gender, atopic disease in the parents, and antibiotic therapy in pregnancy. * Dairy products including butter and cream

Supplementary Table S5. Sensitivity analysis of controls by transient AD status

	**Atopic dermatitis (twice until 2 years) versus control with no AD at least twice**		**Atopic dermatitis (twice until 2 years) versus control (no AD)**	
	AD in n=27 of 116 (23.3%)		AD in n=27 of 90 (30%)	
	**OR (95% CI)**	***p-value***	**OR (95% CI)**	***p-value***
**Maternal diet**				
**Macronutrients %TEI**				
Total protein	0.60 (0.04–9.31)	*0.714*	1.03 (0.82–1.28)	*0.824*
Ratio vegetal to animal protein	0.96 (0.16–4.53)	*0.958*	0.58 (0.09–2.89)	*0.530*
Total carbohydrates	0.69 (0.04–13.66)	*0.805*	0.97 (0.9–1.03)	*0.324*
Total fats	1.43 (0.09–25.88)	*0.803*	1.05 (0.97–1.14)	*0.268*
Saturated fat (SFA)	1.04 (0.88–1.23)	*0.655*	1.09 (0.9–1.32)	*0.386*
Monounsaturated fat (MUFA)	1.03 (0.89–1.18)	*0.696*	1.09 (0.93–1.27)	*0.285*
Polyunsaturated fat (PUFA)	0.93 (0.61–1.38)	*0.738*	1.06 (0.68–1.62)	*0.781*
**Fiber and Micronutrients**				
Total fibre, gr	1.36 (0.32–6.12)	*0.678*	1.51 (0.34–7.57)	*0.597*
Iron, mg	3.25 (0.16–83.36)	*0.458*	1.35 (0.84–2.24)	*0.226*
Retinol	1.00 (1.00–1.01)	*0.220*	1.0 (1.0–1.0)	*0.318*
Vitamin D	1.14 (0.54–2.42)	*0.733*	1.36 (0.58–3.27)	*0.485*
Daily multivitamins	2.47 (0.84–8.56)	*0.121*	2.27 (0.73–8.17)	*0.177*
Sweetened drinks, mL	0.95 (0.73–1.25)	*0.698*	0.93 (0.7–1.26)	*0.631*
Water consumption, mL	1.01 (0.66–1.81)	*0.967*	0.97 (0.53–1.77)	*0.905*
**Food groups**				
Fruit portions / day	1.12 (0.34–3.48)	*0.849*	1.25 (0.36–4.39)	*0.724*
Vegetable portions / day	0.79 (0.17–3.62)	*0.766*	1.2 (0.24–6.45)	*0.824*
Any nut and soy consumption	1.96 (0.59–6.20)	*0.255*	1.77 (0.5–6.07)	*0.363*
Quantity of red meat, g/day	1.10 (0.73–1.77)	*0.668*	1.31 (0.85–2.19)	*0.257*
Quantity of white meat, g/day	0.67 (0.41–1.07)	*0.094*	0.74 (0.44–1.21)	*0.230*
Fish, g/day	0.80 (0.55–1.18)	*0.206*	0.81 (0.53–1.22)	*0.312*
Dairy products*	0.55 (0.10–3.17)	*0.502*	0.74 (0.12–4.91)	*0.748*
Mediterranean diet score in pregnancy (above vs. below median)	0.24 (0.08–0.69)	***0.009***	0.25 (0.08–0.79)	***0.020***
Number of food items (above vs. below median)	0.19 (0.06–0.58)	***0.005***	0.24 (0.06–0.81)	***0.026***
Maternal Diet Index (above vs. below median)	1.08 (0.38–3.06)	*0.887*	1.47 (0.47–4.68)	*0.505*
Plant-based Index (above vs. below median)	1.05 (0.37–3.04)	*0.921*	0.87 (0.29–2.64)	*0.797*

Regression models are adjusted for total energy intake, gender, atopic disease in the parents, and antibiotic therapy in pregnancy.

* Dairy products including butter and cream

Supplementary Table S6. PCA for individual scores, and association with atopic dermatitis in the child

Pattern	OR	95% CI	*adjusted p-value*
**Atopic dermatitis up to 2 years**			
Pattern 1: healthy, high-fat dairy	0.89	0.67, 1.21	*0.435*
Pattern 2: red-meat, low plant proteins, sweets	0.98	0.72, 1.35	*0.894*
Pattern 3: dairy, fruits and sweets	1.23	0.89, 1.72	*0.220*
Pattern 4: sweets, processed grains	0.94	0.66, 1.31	*0.705*
Pattern 5: Italian	0.98	0.70, 1.37	*0.913*
**Atopic dermatitis up to 1 year**			
Pattern 1: healthy, high-fat dairy	0.77	0.55, 1.05	*0.087*
Pattern 2: red-meat, low plant proteins, sweets	1.12	0.76, 1.69	*0.578*
Pattern 3: dairy, fruits and sweets	1.06	0.71, 1.59	*0.769*
Pattern 4: sweets, processed grains	0.87	0.58, 1.30	*0.504*
Pattern 5: Italian	1.22	0.81, 1.87	*0.352*
**Atopic dermatitis at 4 months**			
Pattern 1: healthy, high-fat dairy	0.88	0.68, 1.13	*0.312*
Pattern 2: red-meat, low plant proteins, sweets	1.25	0.94, 1.71	*0.150*
Pattern 3: dairy, fruits and sweets	1.04	0.78, 1.39	*0.777*
Pattern 4: sweets, processed grains	1.00	0.74, 1.36	*0.994*
Pattern 5: Italian	0.85	0.62, 1.17	*0.321*

Regression models are adjusted for total energy intake, gender, atopic disease in the parents, and antibiotic therapy in pregnancy.

Supplementary Table S7. Representative variables of a given principal component (pattern)

Pattern 1 "healthy, high-fat dairy"

Food group	*correlation (r)*	*p-value*
Plant-based oils	0.622	*0.000*
Couscous, rice	0.560	*0.000*
Vegetables, carrots	0.551	*0.000*
Salad	0.544	*0.000*
Potatoes, boiled	0.534	*0.000*
High-fat dairy	0.527	*0.000*
Whole bread	0.498	*0.000*
Avocado	0.495	*0.000*
Vegetables, cabbage-like	0.476	*0.000*
Fruits (pineapple, apple, pear, plur grapes)	0.460	*0.000*
Tofu	0.455	*0.000*
Hard cheese	0.451	*0.000*
Eggs, any style	0.442	*0.000*
Vegetables, green beans	0.441	*0.000*
Vegetables, green peas	0.391	*0.000*
Tomato sauce	0.388	*0.000*
Drinks, coffee incl. decaffeinated	0.371	*0.000*
Dairy excl. high-fat	0.304	*0.001*
Soup, vegetable broth	0.303	*0.001*
Pasta, ravioli	0.286	*0.002*
Fruits (oranges, kiwi, peach, apricot	0.283	*0.002*
Mayonnaise	0.261	*0.005*
Cold cereals, corn-flakes	0.256	*0.006*
Drinks, fruit juice	0.253	*0.006*
White meat	0.205	*0.027*
Soft cheese	0.192	*0.039*

Pattern 2 "red-meat, low plant proteins, sweets"

Food group	*correlation (r)*	*p-value*
Red meat, minimally processed	0.608	*0.000*
Red meat, processed	0.576	*0.000*
French fries	0.543	*0.000*
White meat	0.492	*0.000*
Sweets, baked	0.463	*0.000*
Drinks, sweetened (lemonade, soda)	0.457	*0.000*
Drinks, fruit juice	0.392	*0.000*
Sweets (honey, jam, ice-cream, sugar)	0.379	*0.000*
White bread, rusks	0.371	*0.000*
Fruits (berries)	0.267	*0.004*
Yogurt	0.250	*0.007*
Vegetables, tomatoes	0.246	*0.008*
High-fat dairy	0.244	*0.008*
Fruits (oranges, kiwi, peach, apricot)	0.237	*0.010*
Salad	-0.234	*0.011*
Eggs, any style	-0.252	*0.006*
Vegetables, carrots	-0.284	*0.002*
Tofu	-0.345	*0.000*

Pattern 3 "Dairy, fruits and sweets"

Food group	*correlation (r)*	*p-value*
Yogurt	0.536	*0.000*
Fruits (berries)	0.418	*0.000*
Cold cereal, corn-flakes	0.407	*0.000*
Sweets (honey, jam, ice-cream, sugar)	0.332	*0.000*
Soft cheese	0.310	*0.001*
Fruits (pineapple, apple, pear, plum, grapes)	0.298	*0.001*
Fruits (oranges, kiwi, peach, apricot)	0.287	*0.002*
Whole bread	0.270	*0.003*
Drinks, fruit juice	0.260	*0.005*
Vegetables, tomatoes	0.216	*0.020*
Pizza, quiche	0.197	*0.034*
Drinks, sweetened (lemonade, soda)	0.183	*0.049*
Pasta, ravioli	-0.184	*0.048*
Vegetables, green peas	-0.190	*0.041*
Hard cheese	-0.202	*0.030*
Fish and sea food	-0.246	*0.008*
White meat	-0.282	*0.002*

Pattern 4 "Sweets, processed grains"

Food group	*correlation (r)*	*p-value*
Chocolate	0.550	*0.000*
Sweets, baked	0.331	*0.000*
White bread, rusks	0.330	*0.000*
Pizza, quiche	0.262	*0.004*
Sweets (honey, jam, ice-cream, sugar)	0.252	*0.006*
Soup, vegetable broth	0.235	*0.011*
Potatoes, boiled	0.221	*0.017*
Tomato sauce	0.207	*0.026*
Vegetables, green peas	0.189	*0.042*
Salad	-0.192	*0.039*
White meat	-0.263	*0.004*
Fish and sea food	-0.314	*0.001*
Drinks, coffee incl. decaffeinated	-0.345	*0.000*
Fruits (berries)	-0.369	*0.000*
Margarine	-0.418	*0.000*
Dairy excl. high-fat	-0.439	*0.000*
Vegetables, tomatoes	-0.444	*0.000*

Vegetables, cabbage-like	-0.313	*0.001*
Soup, vegetable broth	-0.314	*0.001*
Mayonnaise	-0.323	*0.000*
Red meat, processed	-0.338	*0.000*
Drinks, alcoholic beverages	-0.404	*0.000*
Red meat, minimally processed	-0.494	*0.000*

Pattern 5 "Italian"

Food group	*correlation (r)*	*p-value*
Hard cheese	0.433	*0.000*
Pasta, ravioli	0.394	*0.000*
Pizza, quiche	0.360	*0.000*
Chocolate	0.315	*0.001*
Tomato sauce	0.269	*0.003*
Dairy excl. high-fat	0.265	*0.004*
Drinks, coffee incl. decaffeinated	0.264	*0.004*
Sweets, baked	0.197	*0.034*
Drinks, fruit juice	0.193	*0.038*
Vegetables, carrots	-0.283	*0.002*
Soft cheese	-0.290	*0.002*
Vegetables, tomatoes	-0.392	*0.000*
Fruits (oranges, kiwi, peach, apricot)	-0.407	*0.000*
Vegetables, green peas	-0.438	*0.000*
Potatoes, boiled	-0.470	*0.000*

Supplementary Table S8. Correlation of food groups with PCA derived patterns

	**Pattern 1**<br>***r***	**Pattern 2**<br>***r***	**Pattern 3**<br>***r***	**Pattern 4**<br>***r***	**Pattern 5**<br>***r***
Yogurt	0.114	0.250	0.536	0.150	0.125
Soft cheese	0.192	0.179	0.310	0.072	-0.290
Hard cheese	0.451	0.007	-0.202	-0.178	0.433
High-fat dairy	0.527	0.244	-0.141	0.146	-0.167
Dairy excl. high-fat	0.304	0.031	0.136	-0.439	0.265
Plant-based oils	0.622	-0.092	0.149	-0.181	0.078
Margarine	-0.101	0.135	-0.076	-0.418	-0.044
Mayonnaise	0.261	0.164	-0.323	0.162	-0.123
White bread, rusks	-0.146	0.371	-0.169	0.330	-0.089
Whole bread	0.498	-0.067	0.270	-0.034	-0.142
Musli, corn-flakes	0.256	0.110	0.407	-0.031	0.142
Red meat, minimally processed	0.075	0.608	-0.494	-0.067	0.035
Red meat, processed	0.016	0.576	-0.338	-0.115	0.034
White meat	0.205	0.492	-0.282	-0.263	-0.027
Fish and sea food	0.149	0.167	-0.246	-0.314	0.138
Green vegetables excl. cabbage-like	0.441	-0.042	0.026	-0.052	0.151
Vegetables, cabbage-like	0.476	-0.077	-0.313	-0.162	0.015
Vegetables, tomatoes	0.117	0.246	0.216	-0.444	-0.392
Vegetables, yellow	0.551	-0.284	-0.176	-0.055	-0.283
Salad	0.544	-0.234	0.083	-0.192	0.043
Vegetables, green peas	0.391	0.081	-0.190	0.189	-0.438
Avocado	0.495	-0.103	0.038	0.009	0.106
Tomato sauce	0.388	0.178	-0.003	0.207	0.269
Soup, vegetable broth	0.303	-0.131	-0.314	0.235	-0.165

Potatoes, boiled	0.534	0.022	-0.122	0.221	-0.470
French fries	0.067	0.543	-0.033	0.127	-0.165
Pasta, ravioli	0.286	0.125	-0.184	0.164	0.394
Pizza, quiche	0.143	0.180	0.197	0.262	0.360
Couscous, rice	0.560	0.109	0.022	0.023	0.059
Fruits (oranges, kiwi, peach, apricot)	0.283	0.237	0.287	0.044	-0.407
Fruits (pineapple, apple, pear, plum, grapes)	0.460	-0.042	0.298	-0.081	-0.080
Fruits (berries)	-0.083	0.267	0.418	-0.369	-0.180
Eggs, any style	0.442	-0.252	-0.093	0.146	-0.054
Tofu	0.455	-0.345	0.144	0.114	0.087
Sweets (honey, jam, ice-cream, sugar)	0.077	0.379	0.332	0.252	-0.077
Sweets, baked	0.148	0.463	0.172	0.331	0.197
Chocolate	0.156	-0.114	0.072	0.550	0.315
Drinks, unsweetened (tea, water)	0.182	-0.174	-0.024	0.126	-0.042
Drinks, fruit juice	0.253	0.392	0.260	-0.094	0.193
Drinks, coffee incl. decaffeinated	0.371	-0.021	-0.088	-0.345	0.264
Drinks, sweetened (lemonade, soda)	-0.143	0.457	0.183	0.010	0.173
Drinks, alcoholic beverages	0.040	0.055	-0.404	0.017	0.110
